# Polygenic scores for Alzheimer’s disease risk and resilience predict age at onset of amyloid‐β

**DOI:** 10.1002/alz.090024

**Published:** 2025-01-03

**Authors:** Eleanor K. O’Brien, Tenielle Porter, Shane Fernandez, Timothy Cox, Vincent Dore, Pierrick Bourgeat, Benjamin Goudey, James D. Doecke, Colin L. Masters, Christopher C. Rowe, Victor L Villemagne, Carlos Cruchaga, Andrew J. Saykin, Simon M Laws, ADOPIC Consortium (AIBL, ADNI, OASIS)

**Affiliations:** ^1^ Centre for Precision Health, Edith Cowan University, Joondalup, Western Australia Australia; ^2^ Collaborative Genomics and Translation Group, School of Medical and Health Sciences, Edith Cowan University, Joondalup, Western Australia Australia; ^3^ School of Medical and Health Sciences, Edith Cowan University, Joondalup, Western Australia Australia; ^4^ Curtin Medical School, Curtin University, Bentley, Western Australia Australia; ^5^ The Australian e‐Health Research Centre, CSIRO, Parkville, VIC Australia; ^6^ The Australian e‐Health Research Centre, Commonwealth Scientific and Industrial Research Organisation, Melbourne, VIC Australia; ^7^ Department of Molecular Imaging, Austin Health, Melbourne, VIC Australia; ^8^ The Australian e‐Health Research Centre, Commonwealth Scientific and Industrial Research Organisation, Brisbane, QLD Australia; ^9^ Florey Institute of Neuroscience and Mental Health, University of Melbourne, Parkville, VIC Australia; ^10^ ARC Training Centre in Cognitive Computing for Medical Technologies, Parkville, VIC Australia; ^11^ The Australian e‐Health Research Centre, CSIRO, Brisbane, QLD Australia; ^12^ Department of Molecular Imaging & Therapy, Austin Health, Heidelberg, VIC Australia; ^13^ The Florey Institute of Neuroscience and Mental Health, The University of Melbourne, Parkville, VIC Australia; ^14^ University of Pittsburgh, Pittsburgh, PA USA; ^15^ NeuroGenomics & Informatics Center, Washington University School of Medicine, St. Louis, MO USA; ^16^ Washington University in St. Louis, St. Louis, MO USA; ^17^ Department of Radiology and Imaging Sciences, Center for Neuroimaging, School of Medicine, Indiana University, Indianapolis, IN USA; ^18^ Indiana Alzheimer’s Disease Research Center, Indiana University School of Medicine, Indianapolis, IN USA

## Abstract

**Background:**

Genome‐wide association studies (GWAS) have identified numerous genetic variants associated with Alzheimer’s disease (AD) risk, but genetic variation in the onset and progression of AD pathology is less understood. Accumulation of amyloid‐β (Aβ) in the brain is a key pathological hallmark of AD beginning 10 – 20 years prior to cognitive symptoms. We investigated the genetic basis of variation in age at onset (AAO) of brain Aβ by comparing the performance of polygenic scores (PGSs) based on AD risk and resilience with a Aβ‐AAO trait‐specific PGS.

**Method:**

1122 participants from the Alzheimer’s Dementia Onset and Progression in International Cohorts (ADOPIC) study underwent genome‐wide SNP genotyping and assessment of brain Aβ using positron emission tomography (PET) imaging at two or more timepoints. AAO was the age at which participants were estimated to have crossed the 20 centiloid (CL) threshold for high Aβ. We utilised AD risk and resilience GWAS summary statistics and conducted a GWAS for AAO using a cross‐validation approach (10 test‐validation folds). We used PRSice to identify optimal PGSs for Aβ‐AAO for risk (PGS_Risk_), resilience (PGS_Resilience_) and Aβ‐AAO (PGS_AAO_).

**Result:**

PGS_Risk_ and PGS_Resilience_ were both significantly associated with Aβ‐AAO, such that higher PGS_Risk_ and lower PGS_Resilience_ were associated with an earlier Aβ‐AAO. PGS_Risk_ showed the strongest association and explained more variance in Aβ‐AAO than did PGS_AAO_. When stratified by *APOE* ε4 carriage, the strongest genetic risk factor for AD, the association of PGS_Risk_ with Aβ‐AAO was stronger among ε4 non‐carriers, whilst PGS_Resilience_, was more strongly associated with Aβ‐AAO in ε4 carriers.

**Conclusion:**

PGS based on genetic risk and resilience for AD are both significant predictors of the age at which people are estimated to cross the threshold for high brain Aβ burden. Predicting the age at which a person will pass this threshold would enable treatment at an earlier stage, when it may more effectively delay or prevent symptom onset.

